# Extended Stanford Type A (DeBakey Type I) Aortic Dissection From the Carotid to the Femoral Arteries Presenting as Out-of-Hospital Cardiac Arrest With Global Cerebral Infarction: A Case Report

**DOI:** 10.7759/cureus.108819

**Published:** 2026-05-13

**Authors:** Maryam Zaman, Ali Abbas Mankani, Sai Charan Chilumula

**Affiliations:** 1 Emergency Medicine, Bayhealth Hospital, Dover, USA; 2 Internal Medicine, Dow University of Health Sciences, Civil Hospital Karachi, Karachi, PAK

**Keywords:** abdominal aortic aneurysm (aaa), circulatory shock, out of hospital cardiac arrest, systemic hypertension, type a aortic dissection

## Abstract

Aortic dissection (AD) is a rare but serious vascular emergency that requires rapid diagnosis and intervention. We report the case of a 58-year-old male with a history of poorly controlled hypertension and prior abdominal aortic aneurysm who presented with an extensive Stanford Type A AD originating near the aortic arch and propagating throughout the thoracic and abdominal aorta into the bilateral iliac arteries, extending distally to the left common femoral artery. Proximally, the dissection involved the innominate, subclavian, and bilateral carotid arteries, resulting in severely compromised cerebral perfusion.

This case highlights an exceptionally extensive form of AD with near pan-aortic involvement and widespread branch vessel propagation affecting cerebral, visceral, and peripheral circulations. It also underscores the atypical presentation of AD as sudden cardiac arrest without preceding symptoms, emphasizing the need for a high index of suspicion and comprehensive vascular imaging to accurately assess disease extent and guide management.

## Introduction

With an incidence of 5-30 per 1 million individuals each year, aortic dissection (AD) is a rare but catastrophic, life-threatening emergency requiring immediate intervention [1]. It involves a tear in the intimal layer of the aorta, creating a false lumen between the aortic intima and tunica media or adventitia, which may extend proximally and/or distally [2]. Patients typically present with sharp, tearing chest or back pain, mimicking other disorders such as myocardial infarction (MI) or gastric ulcer perforation, thus requiring a high degree of clinical suspicion, complemented by timely diagnostic imaging such as computed tomography angiography (CTA) for prompt diagnosis and intervention. Other signs and symptoms include shortness of breath (SOB), discrepancy in blood pressure between the upper extremities, neurological deficits, syncope, and deteriorating mental status [3,4]. However, asymptomatic cases of AD have also been reported in the literature [5,6]. If untreated, mortality approaches 33% within the first 24 hours and 50% within 48 hours, with most patients unable to survive before presenting to the emergency department (ED) or before a diagnosis is made [7].

There are two main anatomical classifications of AD. The DeBakey system classifies AD into three types: Type I involving all aortic segments, Type II involving only the ascending aorta, and Type III affecting only the descending aorta. On the other hand, the Stanford classification categorizes dissections involving the ascending aorta as Type A, whereas those affecting only the descending aorta are classified as Type B [2]. Dissections involving the ascending aorta are nearly twice as common as those involving the descending aorta [2]. Cases involving additional arteries such as the subclavian, coronary, and renal arteries have also been reported in the literature; however, these are usually limited and localized to a certain extent. We present a case of a massive dissection of the aortic arch extending into the carotid and vertebral arteries and distally into the femoral arteries.

We present a case of extended Stanford Type A (DeBakey Type I) AD from the carotid to the femoral arteries, presenting as out-of-hospital cardiac arrest with global cerebral infarction. This case reflects the importance of maintaining a high degree of clinical suspicion, making a prompt diagnosis, and obtaining imaging to visualize all major vessels, as our case lacked the usual presenting features of AD, including chest pain, back pain, blood pressure discrepancy in the upper extremities, or hypertension [2]. 

## Case presentation

A 58-year-old male with a past medical history of hypertension, hyperlipidemia, abdominal aortic aneurysm, obesity status post gastric sleeve, and arthritis presented to the ED following an out-of-hospital cardiac arrest in November 2025. The patient had been in his usual state of health and was swimming at a local Young Men’s Christian Association when he collapsed shortly after exiting the pool. There were no reported preceding symptoms, including chest pain, dyspnea, or neurologic deficits. Per family, the patient had been maintaining an overall healthy lifestyle but had been non-adherent to antihypertensive medications. Notably, he had attended a routine cardiology visit earlier the same day.

**Figure 1:**
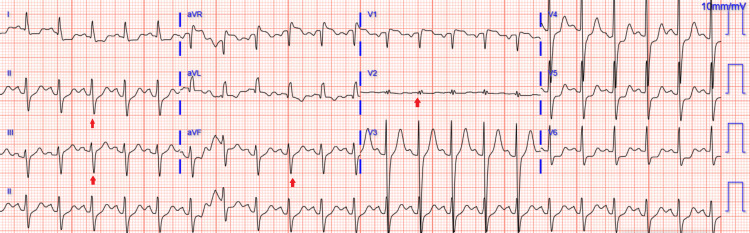
ECG showing RBBB and LAFB. RBBB: right bundle branch block; LAFB: left anterior fascicular block

Cardiopulmonary resuscitation (CPR) was initiated immediately, and return of spontaneous circulation was achieved after two rounds of resuscitation. An I-gel airway was placed by emergency medical services (EMS) in the field and switched to an endotracheal tube in the ED, where he arrived 45 minutes after symptom onset. On arrival, vital signs were notable for a heart rate of 118 beats per minute and blood pressure of 112/78 mmHg, which declined to 72/48 mmHg, requiring vasopressor support with norepinephrine infusion for undifferentiated shock. The patient remained unresponsive with a Glasgow Coma Scale (GCS) of 3 without any sedation administered. Physical examination was remarkable for weak but equal radial pulses, dilated but equal non-reactive pupils, absent gag reflex, and right thigh swelling. A bedside ultrasound demonstrated evidence of bilateral carotid artery dissection. Initial laboratory evaluation demonstrated a high-sensitivity troponin of 13 ng/L (0-14 ng/L), lactate of 1.7 mmol/L (0.5-2.2 mmol/L), and creatinine of 1.5 mg/dL (baseline 1.11 mg/dL) (Table 1). Arterial blood gas analysis revealed a pH of 7.406 (7.35-7.45) (Table 2). ECG showed sinus tachycardia with right bundle branch block and left anterior fascicular block (Figure 1).

**Table 1:** Initial laboratory results upon presentation. MCV: mean corpuscular volume; MCHC: mean corpuscular hemoglobin concentration; RDW: red blood cell distribution width; MPV: mean platelet volume; BUN: blood urea nitrogen; GFR: glomerular filtration rate; ALT: alanine transaminase; AST: aspartate aminotransferase

Lab	Results	Reference
WBC	16.5 × 10³/µL	4.0-11.0 × 10³/µL
RBC Count	4.61 × 10⁶/µL	4.5-5.9 × 10⁶/µL
Hemoglobin	12.7 g/dL	13.5-17.5 g/dL
Hematocrit	38.4%	41-53%
MCV	83.3 fL	80-100 fL
MCH	27.5 pg	27-33 pg
MCHC	33.1 g/dL	32-36 g/dL
RDW	14.2%	11.5-14.5%
Platelets	218 × 10³/µL	150-450 × 10³/µL
MPV	9.8 fL	7.5-11.5 fL
High-Sensitivity Troponin	13 ng/L	0-14 ng/L
Magnesium	1.8 mg/dL	1.7-2.2 mg/dL
Glucose	131 mg/dL	80-140 mg/dL
BUN	21 mg/dL	7-20 mg/dL
Creatinine	1.5 mg/dL	0.7-1.3 mg/dL
eGFR	40 mL/min/1.73 m²	≥60 mL/min/1.73 m²
BUN/Creatinine Ratio	14.0	10-20
Sodium	136 mmol/L	136-145 mmol/L
Potassium	4.4 mmol/L	3.5-5.1 mmol/L
Chloride	98 mmol/L	98-107 mmol/L
CO₂	28 mmol/L	22-29 mmol/L
Anion Gap	10	8-16
Calculated Osmolality	276.7 mOsm/kg	275-295 mOsm/kg
Corrected Calcium	9.5 mg/dL	8.6-10.2 mg/dL
Total Bilirubin	0.7 mg/dL	0.1-1.2 mg/dL
ALT (SGPT)	46 U/L	7-56 U/L
AST (SGOT)	76 U/L	10-40 U/L
Alkaline Phosphatase	63 U/L	44-147 U/L
Total Protein	7.0 g/dL	6.0-8.3 g/dL
Albumin	4.0 g/dL	3.5-5.0 g/dL
Lactate	1.7 mmol/L	0.5-2.2 mmol/L

**Table 2:** Initial arterial blood gases (ABGs) upon presentation.

Lab	Results	Reference
pH	7.406	7.35-7.45
pCO₂	42.7 mmHg	35-45 mmHg
pO₂	474.6 mmHg	80-100 mmHg
HCO₃⁻	26.8 mmol/L	22-26 mmol/L

**Figure 2:**
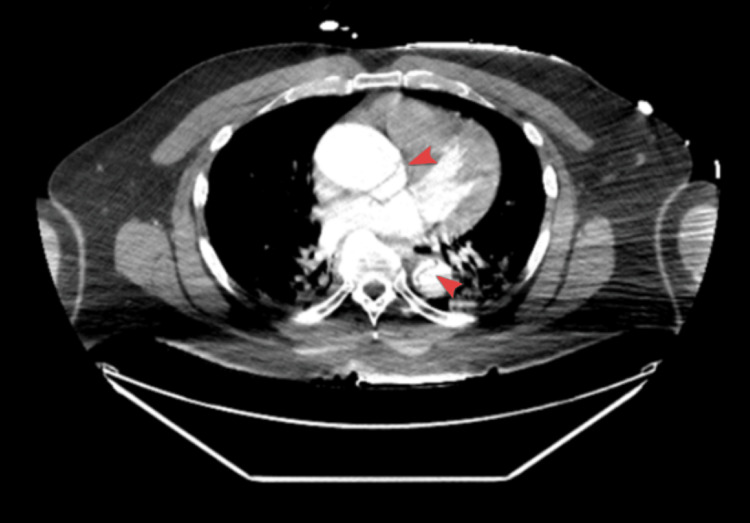
Aortic dissection involving ascending and descending aorta (arrowheads) with possible aortic root involvement.

**Figure 3:**
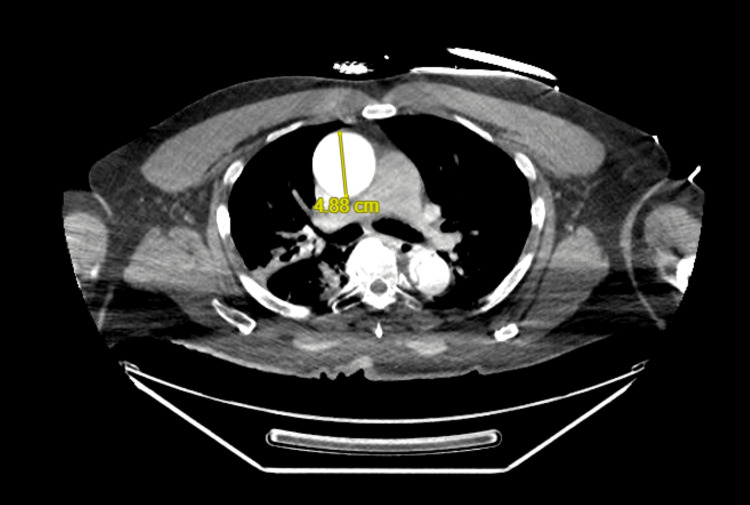
Aneurysmal dilatation of thoracic aorta.

CTA of the chest, abdomen, and pelvis revealed a complex Stanford type A (DeBakey type I) AD originating near the aortic arch, with possible involvement of the aortic root (Figure 2). The dissection extended throughout the entire length of the thoracic and abdominal aorta into the bilateral common and external iliac arteries, terminating on the right in the proximal external iliac artery but continuing through to the left common femoral artery. Proximally, the dissection involved the innominate artery, right subclavian artery, and bilateral common carotid arteries, with poor opacification of the left common carotid artery and left subclavian artery, suggesting compromised flow. The dissection flap further extended into major abdominal branches, including the celiac trunk and superior mesenteric artery. The renal arteries demonstrated differential perfusion, with one arising from the true lumen and the other from the false lumen. There was aneurysmal dilation of the ascending aorta measuring up to 4.88 cm (Figure 3). No pericardial effusion or cardiac tamponade was identified.

**Figure 4:**
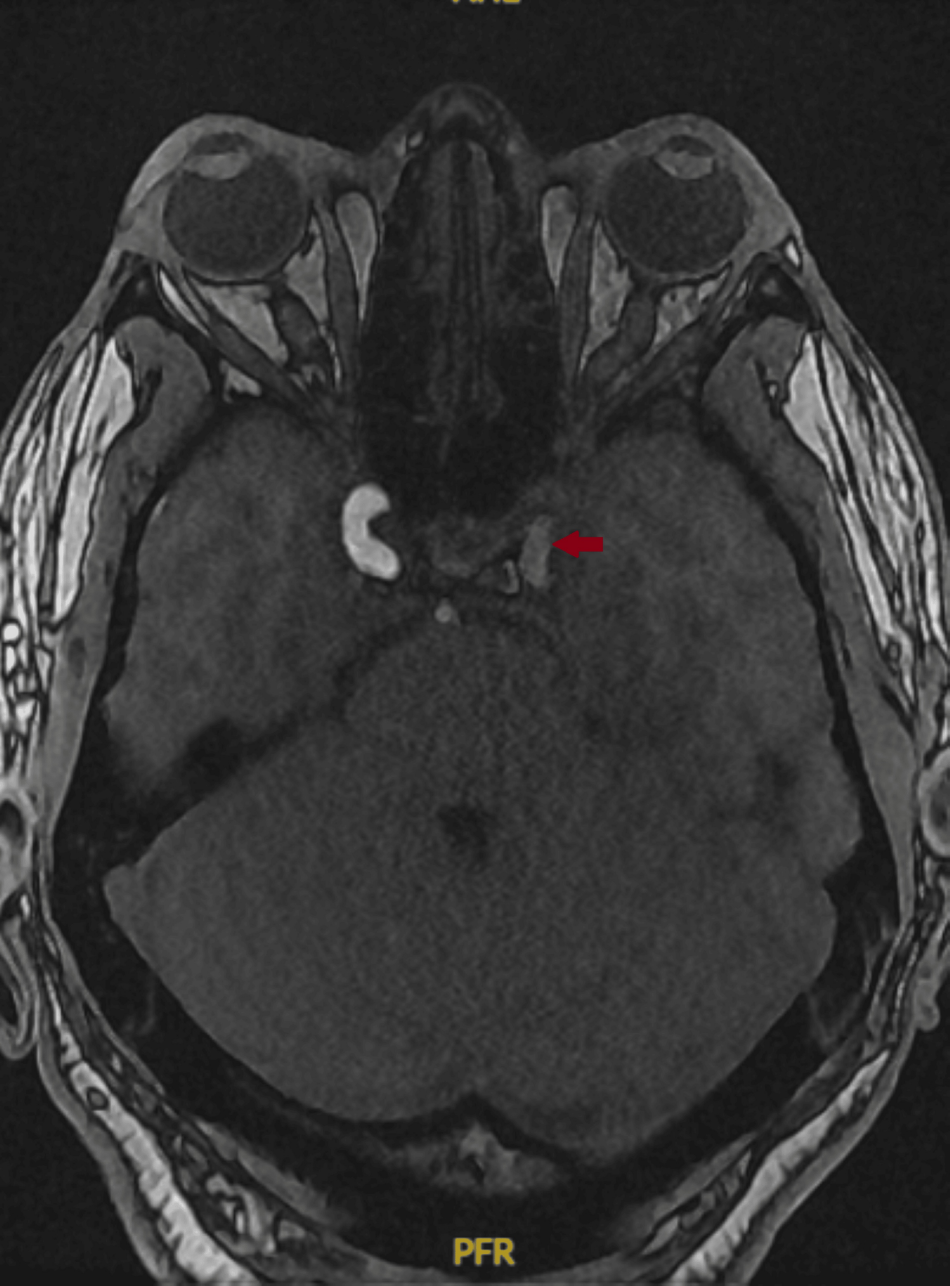
Loss of signal/flow in the left internal carotid artery (arrow).

Magnetic resonance imaging (MRI) of the brain demonstrated extensive early acute infarctions involving the bilateral temporal and occipital lobes, cerebellar hemispheres, brainstem, hippocampi, and thalami, with additional punctate infarcts in the left frontal and parietal lobes. Vascular imaging revealed loss of flow-related signal in the left vertebral artery, basilar artery, bilateral superior cerebellar arteries, bilateral posterior cerebral arteries, as well as the left middle cerebral artery and internal carotid artery (Figure 4), consistent with multifocal high-grade stenosis or occlusion.

The patient was then admitted to the intensive care unit (ICU) for further management. Of note, beta-blockers and vasodilatory agents were held due to circulatory shock requiring vasopressor support. On hospital day 1, neurologic examination revealed a GCS score of 3, with the absence of cough, gag, and corneal reflexes, although spontaneous respirations were preserved. Hemodynamically, the patient remained in sinus rhythm with heart rates in the 90s and systolic blood pressures in the 140s, exceeding target parameters for AD management. Vasoactive support included vasopressin infusion, initiation of phenylephrine, and gradual weaning of norepinephrine to minimize chronotropic effects while maintaining perfusion. The patient developed worsening renal function, shock liver, and metabolic acidosis, for which a bicarbonate infusion was initiated.

Given the extent of the dissection and severe global cerebral ischemia with loss of brainstem reflexes, the patient was deemed not a surgical candidate. After discussions with the family regarding prognosis, goals of care were transitioned to comfort measures. The patient expired two days after admission.

## Discussion

Several risk factors are associated with the development of AD, with chronic hypertension being by far the most common risk factor, accounting for approximately 62%-73% of patients with AD. Other well-documented risk factors include connective tissue diseases such as Marfan syndrome or Ehlers-Danlos syndrome, Turner syndrome, cocaine abuse, traumatic injuries, and diseases involving the aorta, such as coarctation of the aorta or a bicuspid aortic valve [1]. In our case, the history of chronic hypertension likely contributed significantly to the development of AD.

While many cases of spontaneous AD have been reported in the literature, AD propagating throughout the entire thoracic and abdominal aorta into the bilateral common and external iliac arteries, and even reaching the left common femoral artery, is an extremely rare entity. A few case reports of spontaneous AD extending into the bilateral common carotid and iliac arteries can be found in the existing literature, but none to the same extent of aortic involvement as in our patient. Lee et al. reported a case of a 61-year-old male with an extensive AD involving the bilateral common carotid arteries; the ascending, thoracic, and abdominal aorta; and the bifurcation of the right common iliac artery [4]. Similarly, Yeh et al. and Demiryoguran et al. described cases of a 56-year-old and a 63-year-old female, respectively, who presented with spontaneous AD involving the aortic arch, ascending aorta, and bilateral common carotid arteries [8,9]. In both cases, similar to ours, Doppler ultrasound of the carotid arteries played a key role in establishing the diagnosis. Fan et al. also reported the case of a 34-year-old male with AD extending proximally to the carotid arteries and distally to the infrarenal abdominal aorta [10].

ADs that extend beyond the primary vessel to involve major arterial branches, including the subclavian, coronary, and renal arteries, have previously been reported in the literature [4,10-12]. While rupture leading to hemorrhage into the pericardial, thoracic, or abdominal cavities is a major cause of mortality in these patients, fatal outcomes may also occur without rupture. In such cases, myocardial ischemia may play a central role and may arise from compromised coronary perfusion due to luminal obstruction by the intimal flap, propagation of thrombus within the false lumen, reduced diastolic filling pressures, or direct extension of the dissection into the coronary circulation [13,14].

Distal vascular involvement has been described in multiple case reports as a feature of spontaneous AD, occurring in roughly one-quarter to one-third of cases [15]. This typically manifests as compromised perfusion of branch vessels due to either extension of the dissection or dislodgment of emboli. In a study by Hughes et al., multiple arterial territories were involved, with numerous major vessels affected across a small cohort of patients, including the carotid, subclavian, celiac, mesenteric, renal, iliac, and femoral circulations [16].

While AD typically presents with tearing chest pain that radiates to the back, our patient collapsed suddenly after exertion without any preceding symptoms [4]. This atypical presentation highlights the broad and often misleading clinical spectrum of AD, particularly when major branch vessels are involved. Rather than localized pain, patients may present with findings that mimic other acute conditions, such as stroke [4]. Neurologic complications are well recognized in AD, occurring in a significant proportion of cases, and may manifest as altered consciousness, coma, or focal deficits due to compromised cerebral perfusion secondary to stroke or cardiac tamponade [17]. In this case, extensive involvement of the carotid and vertebrobasilar circulation likely led to global cerebral ischemia, explaining the patient’s profound neurologic impairment following return of spontaneous circulation. Syncope in AD patients, therefore, reflects catastrophic disruption of cerebral blood flow and has been associated with higher in-hospital mortality compared to AD patients without syncope [17].

Our case underscores the need for comprehensive vascular imaging once a diagnosis of AD is established. While prior reports have described more localized involvement, such as isolated carotid artery extension, our patient demonstrated a far more extensive process, with dissection spanning nearly the entire aorta and extending from the cervical vessels to the femoral circulation. Even in non-traumatic settings, thorough assessment of all major aortic branches is essential to accurately define the full extent of disease, anticipate complications, and guide appropriate medical or surgical decision-making [4]. In our case, the patient did not receive head imaging in the ED, which later revealed extensive cerebral vascular involvement during the hospital admission, indicating a grim prognosis.

Establishing a diagnosis of AD is not an easy task. Approximately 38% of AD cases are not diagnosed on the initial evaluation, particularly in the absence of overt rupture or hemopericardium, where classic symptoms may not be present [1]. In such situations, limiting evaluation to the aorta alone may underestimate the severity of the disease. Careful assessment of branch vessels is critical, as propagation of the dissection into peripheral and end-organ arteries can occur and carries significant clinical consequences [18]. Our case illustrates an extreme form of this phenomenon, with extensive involvement of both central and distal arterial territories, including the carotid, vertebrobasilar, visceral, iliac, and femoral systems, despite no known history of connective tissue or aortic disease. This highlights the importance of maintaining a high index of suspicion and performing comprehensive vascular imaging through modalities such as CT, chest X-ray (CXR), Doppler ultrasonography, and transesophageal echocardiography (TEE) to fully appreciate the extent and lethality of the process.

## Conclusions

The time to diagnosis and treatment is highly important in AD, as delays in intervention can potentially lead to life-threatening outcomes given the rapid nature of disease progression. This case highlights the importance of maintaining a high degree of clinical suspicion, early recognition, and appropriate imaging required to diagnose AD, as well as assess its extent in predicting overall medical management and prognosis in the absence of typical AD symptoms.
